# Shaping of Dendritic Cell Function by the Metabolic Micro-Environment

**DOI:** 10.3389/fendo.2020.00555

**Published:** 2020-08-28

**Authors:** Eline Constance Brombacher, Bart Everts

**Affiliations:** Department of Parasitology, Leiden University Medical Center, Leiden, Netherlands

**Keywords:** dendritic cells, metabolism, nutrient availability, tumor micro-environment, diabetes

## Abstract

Nutrients are required for growth and survival of all cells, but are also crucially involved in cell fate determination of many cell types, including immune cells. There is a growing appreciation that the metabolic micro-environment also plays a major role in shaping the functional properties of dendritic cells (DCs). Under pathological conditions nutrient availability can range from a very restricted supply, such as seen in a tumor micro-environment, to an overabundance of nutrients found in for example obese adipose tissue. In this review we will discuss what is currently known about the metabolic requirements for DC differentiation and immunogenicity and compare that to how function and fate of DCs under pathological conditions can be affected by alterations in environmental levels of carbohydrates, lipids and amino acids as well as by other metabolic cues, including availability of oxygen, redox homeostasis and lactate levels. Many of these insights have been generated using *in vitro* model systems, which have revealed highly diverse effects of different metabolic cues on DC function. However, they also stress the importance of shifting toward more physiologically relevant experimental settings to be able to fully delineate the role of the metabolic surroundings in its full complexity in shaping the functional properties of DCs in health and disease.

## Introduction

DCs are crucial for the regulation of immunity and tolerance during infections as well as during immune homeostasis in steady state, by governing the activation and maintenance of different CD4^+^ T helper subsets and CD8^+^ cytotoxic T cell responses. DCs are a heterogenic population of cells that comprise several lineages, including conventional DCs (cDCs), plasmacytoid DCs (pDCs), Langerhans cells (LCs), and inflammatory monocyte-derived DCs (infDCs) (1). Two main lineages can be identified within the cDCs, IRF8-dependent cDC1s and IRF4-dependent cDC2s (2). These different DC subsets have specialized largely non-redundant functions in priming and regulation of T cell responses (2). For instance, cDC1s and cDC2s are considered to be the most potent activators of T cells, that are particularly well-equipped to prime CD8^+^ and CD4^+^ T cell responses, respectively. pDCs, on the other hand, play an important role in the viral defense by producing large amounts of type I interferons. infDCs develop from monocytes during inflammation and have been described to have a role in innate inflammatory responses as well as in T cell priming (3). Given the scarcity of DCs *in vivo*, several *in vitro* experimental models have been developed to study DC biology. In this respect monocyte-derived DCs (moDCs), obtained from monocyte cultures supplemented with granulocyte-macrophage colony-stimulating factor (GM-CSF) and IL-4 are a widely used model to study human DC biology and metabolism (4). moDCs share phenotypical and functional similarities with cDCs, but closely resemble infDCs at the transcriptional level (5, 6). *In vitro* generated murine DCs are commonly derived from GM-CSF-stimulated bone marrow (BMDCs), which bear resemblance to antigen presenting monocyte-derived murine inflammatory DCs. Flt3L-stimulated bone marrow cultures (Flt3L-DCs) give rise to equivalents of multiple steady state splenic DC subsets, including cDC1s, cDC2s, and pDCs (7–9). Regulation of the functional properties of DCs is dictated by their ontogeny, but also strongly influenced by their micro-environment. While the effects of danger signals, cytokines and chemokines are extensively reviewed elsewhere, we will focus on the role of metabolic cues in tuning DC function (10–12).

Over the last decade it has become increasingly clear that immune cell activation, proliferation, fate and function are closely linked to, and dependent on activation of specific metabolic pathways (13). Since these metabolic shifts are largely fueled and dependent on nutrients present in the immune cell niche it is becoming evident that the metabolic micro-environment is a vital factor in shaping the outcome of an immune response (14, 15). This review describes the latest insights into the nutritional requirements for DCs to drive an effective immune response and how altered nutrient availability in diseased states may affect the immunogenic or tolerogenic properties of DCs. In this context we will particularly focus on nutrient-limiting and excessive nutrient conditions as found in cancer and diabetes, respectively. Overall we here aim to provide an overview of how the metabolic micro-environment affects the functional properties of DCs.

## Metabolic Demands of DCs During an Immune Response

During homeostasis DCs reside in peripheral tissues in a relatively quiescent state. However, upon sensing of changes in this homeostatic state, either due to invading pathogens or tissue-derived inflammatory signals, DCs undergo a well-defined activation program that involves increased capturing and processing of antigens for presentation in major histocompatibility complex I (MHC-I) and MHC-II and the induction of expression of chemokine receptors, cytokines, and costimulatory molecules. This enables DCs to traffic, via tissue-draining lymphatic vessels, to T cell zones of secondary lymphoid organs to efficiently prime and control effector T cell responses. Here, we discuss how the availability of carbohydrates (glucose), amino acids and lipids influences these functions of DCs during an immune response.

### Glucose

**Figure 1:**
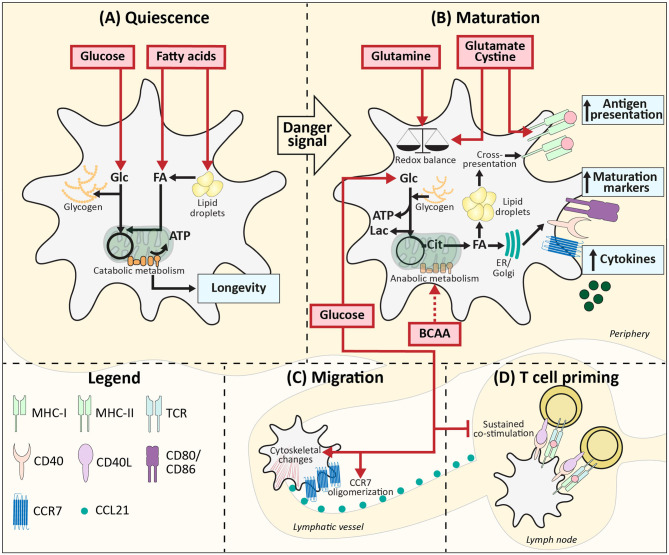
Metabolic demands of conventional DCs during homeostasis. **(A)** Quiescent DCs in peripheral tissues require, glucose and lipids as fuel for mitochondrial ATP generation and to build up intracellular storage of lipids and glycogen. **(B)** After TLR ligation uptake of glucose and BCAAs increases and together with glucose derived from glycogen this supports the switch from catabolic to anabolic metabolism, which is required for DC maturation. During this process, lipid bodies support cross-presentation. Glutamine, cysteine, and glutamate promote DC activation via maintaining redox homeostasis and promoting antigen presentation. **(C)** Glucose also promotes migration via stimulating CCR7 oligomerization and inducing cytoskeletal changes. **(D)** In the lymph nodes local glucose availability may be reduced due to glucose consumption by T cells, which may allow for more sustained expression of costimulatory markers and thereby more potent T cell priming. Red boxes: nutrients. Blue boxes: Functional consequences. Dotted arrow: presumed mechanism. ER, Endoplasmatic reticulum; Golgi, Golgi apparatus.

A well-described phenomenon in immune cells, including DCs, is the switch from catabolic metabolism, characterized by fatty acid oxidation and mitochondrial respiration (Figure 1A) to anabolic metabolism, with enhanced glycolytic rates and lowered oxidative phosphorylation following activation (Figure 1B). BMDCs increase glycolytic rates within minutes after stimulation with LPS (TLR4), Poly(I:C) (TLR3), and CPG (TLR9) and also for moDCs a rapid glycolytic increase has been observed upon LPS stimulation (16–20). Likewise, human myeloid CD1c^+^ DCs and human pDCs show a similar metabolic reprogramming upon pRNA (TLR7/8) stimulation or viral infection (TLR7), respectively (21, 22). Although this points toward increased glucose utilization to fuel glycolysis as a conserved metabolic response to TLR stimulation by DCs, the function of this metabolic reprogramming and fate of glucose-derived carbons can change over time after activation, as discussed below.

For LPS-induced BMDCs the switch to enhanced glycolysis is essential, given that inhibition of glycolysis using 2-deoxyglucose (2-DG) interferes with activation and their capacity to induce T cell proliferation *in vitro* and *in vivo* (16, 23). Correspondingly, changing glucose concentrations in culture media from 10 to 0 mM diminished the upregulation of co-stimulatory markers CD40 and CD86, and production of p40, subunit of IL-12 and IL-23 in LPS-stimulated murine BMDCs (16). In the presence of glucose, LPS stimulation enhances glycolysis via activation of the PI3K/Akt pathway and promotes loss of mitochondrial respiration via stabilization of Hypoxia-inducible factor 1-alpha (HIF1α) and expression of Inducible nitric oxide synthase (iNOS) in BMDCs (16, 17, 24). iNOS is involved in production of nitric oxide (NO) which in an autocrine manner poisons oxidative phosphorylation (17, 24). These TLR-induced events are largely absent in BMDCs activated by LPS in the absence of glucose, due to impaired mTOR activation, thereby further establishing the crucial role for glucose in supporting TLR-induced DC activation (Figure 1B). Mechanistically, BMDCs require glucose as a carbon source for fatty acid synthesis (FAS) to support ER and Golgi membrane expansion that is needed to accommodate the increased demands for protein translation during activation (23). To this end, glucose is metabolized in the TCA cycle to citrate, which is used as a carbon substrate for FAS (Figure 1B). In contrast to this anabolic role that glucose-derived carbon plays during BMDC activation, once activated, glucose is used in glycolysis by BMDCs purely for bioenergetic purposes (e.g., synthesis of ATP) to compensate for loss of OXPHOS due to the autocrine effects of NO derived from iNOS that poisons the ETC (17). Apart from direct usage of extracellular glucose, BMDCs and moDCs can also utilize intracellular glycogen stores to fuel glycolysis. These glycogen stores are formed prior to TLR stimulation and essential for activation and subsequent T cell stimulation in both moDCs and BMDCs by directly contributing to FAS (Figures 1A,B) (19).

Upon TLR-ligation, DCs upregulate CCR7 and are attracted toward lymphatic vessels that secrete CCR7 ligand CCL21 (25). In BMDCs the presence of glucose in the medium and subsequent activation of glycolysis are required for CCR7 oligomerization and cytoskeletal changes that support the mobility of DCs. Correspondingly, glucose depletion reduced migration of splenic DCs toward CCL21 *ex vivo* and 2-DG administration in an experimental model of allergic asthma, reduced migration of CD11c^+^MHCIIhi DCs to the lung (26). Similarly, BMDCs pulsed *ex vivo* with OVA plus LPS in the presence of 2-DG, displayed impaired migration toward skin-draining lymph nodes following subcutaneous injection. Altogether this points toward an important role for glucose in DC migration *in vivo* (Figure 1C) (16).

Interestingly, there are indications that the role of glucose in priming of T cell responses by DCs changes once the cells interact with T cells. It has been shown that the T cell-priming capacity of BMDCs declines after 24 h following TLR stimulation, which was associated with a reduction in expression of costimulatory molecules (27). However, this reduction in expression was prevented when 8 h after stimulation glucose was replaced with galactose, a carbohydrate that cannot be efficiently used in glycolysis. These data indicate that after the initial need for glucose, sustained high glycolytic rates repress BMDC activation (Figure 1D). Hence a local glucose-limiting micro-environment when DCs interact with T cells, may actually support T cell priming and an active immune response (24). Interestingly, both *in vitro* and *in vivo* data suggest that reduction of glucose availability may occur naturally during T cell priming in lymph nodes, due to scavenging of glucose by T cells that are being activated by DCs (24).

Many studies addressing the role of glycolysis, use glucose analog 2-DG to mimic glucose starvation. 2-DG can reduce mitochondrial metabolism independently of reducing glycolysis (28). Moreover, treatment of LPS-activated moDCs with 2-DG has been documented to result in ER-induced upregulation of IL-23 expression, while glucose depletion did not. These data indicate that the effects of 2-DG are not always connected to glycolysis and warrant caution when interpreting data from studies that have used 2-DG to interrogate the role of glycolysis in DC biology (29). In addition, most of the studies described above have been performed in BMDCs, in which iNOS plays a major role in the suppression of mitochondrial respiration in response to TLR stimulation. Most other DC subsets, including moDCs and primary human DCs, do not express iNOS (30). Hence, the role of oxidative phosphorylation during an immune response in human DCs may be underestimated and in physiological settings DCs may rely less on glucose as predicted based on BMDC experiments. Thus far, it has not been addressed whether other nutritional carbohydrates shape DC function, but it is worth investigating, given that an immunosuppressive role for D-mannose was found in T cells (31). In conclusion, there is clear evidence that many aspects of DC activation are dependent on availability of glucose in the micro-environment. Glucose initiates metabolic reprogramming required for activation and boosts DC migration toward lymph nodes, while during T cell interaction glucose may have an immunosuppressive effect on DCs.

### Amino Acids

Amino acids are important for fueling mitochondrial respiration, for protein synthesis, as well as acting as a source of carbon and nitrogen for the synthesis of various other macromolecules. There are clear indications that amino acids in the environment of DCs play an important role in regulating their function. For example, lowering the supraphysiological amino acid concentrations commonly found in standard culture media to ones found in human plasma, increased the efficiency of moDC differentiation (32, 33). Conversely, moDCs in media containing imbalanced amino acid concentrations, as found in plasma of liver cirrhosis patients, showed impaired expression of maturation markers, secretion of IL-12 and migratory capacity in response to LPS stimulation, compared to moDCs stimulated in control medium (32, 33). The amino acid imbalance interfered with mitochondrial metabolism of immature DCs, causing a reduction in ATP levels and an increase in glucose consumption, which could not be further increased by LPS stimulation. This together supports the idea that several aspects of DC biology, including differentiation, activation and core metabolism, are sensitive to changes in amino acid concentrations in their environment (33).

In addition to the aforementioned work implicating amino acids in general in regulating DC function, there are several studies that have specifically interrogated the role of individual amino acids in this context. LPS stimulation of moDCs has been shown to enhance the uptake of aspartate, cystine, glutamate, and branched chain amino acids (BCAAs) valine, leucine, and isoleucine (33). Depletion of BCAAs and in particular valine from culture media of moDCs impairs maturation upon LPS stimulation, characterized by lowered CD83 levels and decreased ability to induce T cell proliferation. Additionally, mTOR signaling was impaired, which raises the possibility that BCAAs may affect DC maturation through modulation of metabolism via the mTOR pathway (Figure 1B) (34). Of note, as the above mentioned studies were performed in serum-free media with high glucose concentrations (25 mM), the relevance of these results under more physiological settings remains to be established. BCAAs are also important for maturation of moDCs stimulated with TLR7/8 ligand protamine-RNA (pRNA). In contrast to LPS stimulation, pRNA ligation boosts fatty acid oxidation (FAO)-dependent mitochondrial respiration and high intracellular levels of BCAAs are required to induce moDC maturation via FAO (20). BCAA leucine may play a key role in supporting FAO, as leucine can promote mitochondrial biogenesis (35). LPS treatment also increases uptake of glutamate and cystine in moDCs and inhibition of the cystine/glutamate antiporter in these cells reduced glutathione synthesis, but did not change the expression of maturation markers. Nevertheless, treating murine splenic DCs with an cystine/glutamate antiporter inhibitor resulted in lowered antigen presentation to both class I and class II MHC-restricted T cells (Figure 1B) (36). Hence, cystine and glutamate may be crucial metabolites for DC maturation via their role in redox homeostasis and antigen presentation.

As mentioned before, FAS is upregulated in BMDCs following LPS-stimulation. In cancer cells glutamine can contribute to lipogenesis via NADPH production that takes place when glutamine is metabolized to lactate or when glutamine is converted to citrate, facilitated by reductive carboxylation (37, 38). However, depleting glutamine (from 2 to 0 mM) from BMDC culture media did not affect CD40 and CD86 levels and inhibition of glutaminolysis had no effect on the metabolic alterations 6 h after LPS stimulation (19, 23). Interestingly, in BMDCs stimulated with TLR7/8 ligand imiquimod, glutamine deprivation, or disruption of glutaminolysis enhanced mitochondrial reactive oxygen species (ROS) production and subsequently IL-23 expression. This may suggest that glutamine, by supporting NADPH production, may contribute to scavenging of ROS in BMDCs, rather than to FAS-dependent activation (Figure 1B) (39). In addition, glutamine may fuel the TCA cycle to support oxidative phosphorylation in DCs as human pDCs increased oxidative phosphorylation following pRNA stimulation and inhibition of glutaminolysis in these cells caused a significant decrease in activation, IFNα secretion and mitochondrial respiration (21). Since activation of human CD1c^+^ myeloid DCs using pRNA resulted in reduced oxidative phosphorylation and as immunogenicity remained unaffected by inhibition of glutaminolysis, this effect of glutamine may be selectively associated with DC subsets that depend on mitochondrial respiration upon activation, such as pDCs (21).

Apart from glutamine, the importance of availability of different amino acids on DC biology are still poorly defined and are mainly addressed in moDCs. Possibly, due to the low proliferative capacity of differentiated DCs and therefore expected relative little dependency on anabolic metabolism, general amino acid availability may be less of critical factor for DC function than for other more proliferating cells. Nonetheless, as studies exploring the role of glutamine on DC function suggest, specific amino acids may be important in regulation of certain metabolic properties of DCs that are essential for their functional output. Hence, single amino acid depletion studies under more physiological nutrient levels may unravel novel roles of amino acids in DC function.

### Lipids

In contrast to activated DCs, in which glycolysis is often the main bioenergetic pathway, immature quiescent BMDCs and Flt3L-induced cDC1s rely on FAO for energy generation, which would support a longer lifespan for these immature cells (Figure 1A) (16, 17, 40). Lipids from the local micro-environment may function as important nutrients for FAO in resting DCs. In human Lin-HLA-DR^+^ and murine CD11c^+^ hepatic DCs, high lipid content is associated with a stronger immune response (41). These lipids derived from both fatty acid (FA) uptake and synthesis and are stored in lipid bodies. Short term priming with triacyl glycerides of murine DCs containing few lipid bodies did not boost their immunogenic capacities, suggesting that pre-stored lipids rather than direct lipid availability in the micro-environment is important for hepatic DC immunogenicity (41). Mechanistically, lipid bodies in murine BMDCs and splenic CD11c^+^ DCs have been shown to boost CD8^+^ T cell priming by supporting cross-presentation, a process by which peptides from exogenous antigens are presented in MHC-I (42). It is therefore tempting to speculate that resting DCs may not only utilize FAs from the extracellular environment to fuel FAO for their bioenergetic homeostasis, but also to form lipid bodies to help prepare them for potent T cell priming after activation (Figure 1A).

In contract to conventional DCs, FAO can increase upon TLR7/8/9 stimulation of pDCs (20, 43). Interestingly, FAO in murine bone marrow-derived pDCs is fueled with FAs that are synthesized *de novo* (43). LPS is also known to increase FAS in BMDCs, possibly suggesting that FAO during an immune response predominantly depends on *de novo* synthesis and not on the FA availability in the micro-environment (Figure 1B) (18, 23). Correspondingly, in rats it was found that lipid content between different cell types in the same micro-environment was more similar than lipid content between DCs from distinct lymph nodes. Additionally, *in vivo* LPS stimulation diminished the differences observed between distinct DCs, supporting the notion that lipid accumulation during inflammation is independent of FA availability, while lipid storage during homeostasis does appear to be determined by the micro-environment (Figure 1A) (44).

In summary, it appears that during homeostasis lipid availability influences the types and amount of lipids stored in DCs and at least in some tissues this is important for their immunogenic potential. During an immune response, both conventional and pDCs accumulate lipids, most likely independent of the FA availability in the local micro-environment, but fueled by FAS. For cDCs the reduced ability of activated DCs to burn FAs by FAO may also contribute to lipid accumulation (16). Cultures in lipid-restricted conditions, ^13^C-labeled lipid metabolic flux analysis and lipid profiling of DCs during homeostasis and upon activation can further elucidate the role of extracellular lipids on DC function.

### Concluding Remarks

The metabolic demands of DCs in non-pathological conditions are dependent on the subset, their location and the maturation stage, as summarized in Figure 1. Given that most of these data are obtained from *in vitro* studies it is important to realize that *in vitro* nutrient availability is often not limiting and exceed the levels that occur *in vivo*. Furthermore, nutrient competition with cells in the proximity of DCs and metabolites secreted by these surrounding cells are metabolic settings that are hard to mimic *in vitro* and difficult to measure *in vivo*, but likely to affect the metabolic micro-environment. Nevertheless, it is evident that nutrient availability is of great importance for the functional output by DCs.

## Effects of the Metabolic Environment on DCs in Cancer

### Metabolic Properties of the Tumor Micro-Environment

Disturbance of nutrient homeostasis is a cause and consequence of many pathologies. A well-studied and complex disease is cancer, which is characterized by a wide range of local metabolic alterations, including nutrient deficiency, hypoxia and oxidative stress. Cancer is a heterogenous disease that arises from cells with traits that allow uncontrolled proliferation. One of these traits, or hallmarks, is avoiding immune detection, required to prevent elimination by the immune system (45). Cells within the tumor micro-environment (TME), including tumor cells, fibroblasts, endothelial cells, and immune cells secrete immunomodulatory signals that regulate the anti-tumor immune response (46). Among these factors are cytokines, growth factors and metabolites. During the initial phases of tumor growth, tumor-associated DCs (TADCs) are able to recognize tumor antigens and initiate an anti-tumor T cell response. However, during tumor progression DCs gain tolerogenic rather than pro-inflammatory properties (47–49). A major contributor to immune suppression and another hallmark of cancer is deregulated cellular metabolism (45). The best known metabolic adaptation of cancer cells is the Warburg effect, the conversion of glucose to lactate under aerobic conditions, which allows for rapid production of ATP and biosynthetic precursors (50). In addition, tumors also use amino acids and lipids to fuel the TCA cycle, which promotes ATP generation via oxidative phosphorylation and synthesis of macromolecules to support cell growth and proliferation (51–53). Another cancer-specific metabolic feature is the accumulation of oncometabolites due to mutations in metabolic enzymes. L- or D-2-hydroxyglutarate (L- or D2-HG) is a well-known oncometabolite that promotes tumor growth by regulating DNA and histone modifying enzymes (54). Finally, malignancies are often characterized by unusually high concentrations of extracellular ATP and adenosine, hypoxia and by large quantities of ROS in poorly vascularized regions (52, 55). The above mentioned metabolic changes and stressors do not only affect tumor cells, but also reach stromal cells residing in the TME. Here we will describe how these metabolic cues affect DCs.

### Nutrient Starvation

The excessive utilization of carbohydrates, amino acids and lipids by cancer cells results in a limited nutrient supply for cells residing in the TME. Although there are several seminal papers showing how nutrient limitation in the TME impairs CD8^+^ T cell function, there are few studies that have directly interrogated the contribution of nutrient starvation to the known suppressive effects of the TME on DCs (56, 57). Initially upon entering of DCs into the TME, one could imagine that TADCs may be able to utilize internal stores of glycogen and lipids to support the metabolic demands for their survival and immunogenic activation (19, 41). However, sustained limited access to glucose may impair metabolic rewiring and thereby DC maturation and migration to tumor draining lymph nodes (16, 24, 26). Likewise, based on *in vitro* studies, as discussed in section Amino Acids, it stands to reason that also insufficient access to amino acids may compromise TADC function by affecting mitochondrial respiration, redox homeostasis and antigen presenting capacity (33, 36, 39). However, to date no studies have directly addressed this. On the other hand, the effects of lipids on the function of TADCs have been studied more extensively and hence will be discussed in a separate section.

Tolerogenic properties of DCs have been linked to increased FAO and mitochondrial respiration (18, 58). These processes are both stimulated by activation of AMPK, an energy-sensing enzyme that is activated under nutrient limiting conditions (59). AMPK has been shown to be inactivated in DCs upon LPS-induced activation (16). Conversely, in TADCs of mice inoculated with MC38 colon adenocarcinoma cells, activation of LKB1, one of the main activating kinases of AMPK, was elevated (60). Hence, the nutrient-poor TME may boost AMPK signaling in DCs to induce catabolic metabolism that favors tolerogenic properties. Although no study to date has examined it directly, it is likely that limited nutrient access in the TME contributes to immune suppression of DCs (Table 1). Addressing the influence of tumors with different bioenergetic profiles on DC activation *in vivo* and *in situ* will provide more insights into the effects of nutrient deprivation on DC-driven immune suppression.

**Table 1:** Metabolic determinants from pathological environments that influence DC function.

**Effects associated with the TME**	**Effects associated with diabetes**	**References**
**Glucose**		(16, 19, 23, 26, 61–71)
Local depletion	Hyperglycemia	
Impaired anabolic metabolism ≫ ↓ Activation ≫ ↓ Migration *AMPK activation* ≫ *↓ Activation*	Blood DCs ↓/↑ DC counts = Activation ↑ *Migration to metabolic tissues* *In vitro* DCs ↓/= Differentiation ↑ Activation ≫ ↑ *Th17 T cell priming*	
**Lipids**		(39, 72–83)
High intracellular storage	Hyperlipidemia	
Oxidized lipids ↓ Cross-presentation ↑ *Fatty acid oxidation* ≫ ↓ Activation	PA ↑ NFκB signaling Metabolic rearrangements ≫ ↑ Activation SA ↓ Activation OA = Activation	
**Oxygen levels**		(84–89)
Low	Low	
= Differentiation		
↑ Innate immunity (immature DCs)		
↑ Migration (immature DCs)		
= /↑/↓ Activation		
↓ Migration (mature DCs)		
**ROS**		(90, 91)
High	High	
Short term ROS		
↑ Activation		
Long term ROS		
*Lipid oxidation*		
≫↓ *Activation*		
**Lactate**		(92–96)
High		
Impaired mitochondrial respiration ≫ ↓ Differentiation ↓ Cross-presentation *Epigenetic modifications* ≫ ↓ Activation		
**ATP and adenosine**		(97–104)
High		
ATP ↑ Migration NLRP3 inflammasome activation ≫ ↑ CD8^+^ T cell priming ↑ regulatory T cell priming Adenosine ↓ Activation		
**2-Hydroxyglutarate**		
High		(105)
↓ IL-12 ↑ Catabolic metabolism ≫ = Activation *Epigenetic modifications*		

*Overview of extracellular metabolic cues observed in cancer and diabetes that affect DC function. ↑/↓/=: increased, decreased or equal effect compared to control conditions. ≫ Consequence of aforementioned mechanism. Italics: presumed effect*.

### Lipid Accumulation in Tumor-Associated DCs

Although tumor cells are generally characterized by high lipid uptake, TADCs can also take up large amounts of FAs from the TME (106). Acquisition of lipids by TADCs is facilitated by the upregulation of genes involved in lipid uptake, including lipoprotein lipase (LPL), fatty acid binding protein 4 (FABP4) and macrophage scavenger receptor (Msr1). The lipids are stored in large lipid droplets, which are associated with a reduced capacity to activate T cells (72–74). As discussed earlier, high lipid content in hepatic DCs is associated with higher immunogenicity and LPS stimulation of BMDCs stimulates lipid droplet formation (23, 41). However, in contrast to lipid droplets of these DCs, lipid droplets from TADCs contain high levels of oxidized polyunsaturated FAs. These oxidized FAs cause accumulation of peptide-bound MHC-I complexes in late endosomes and lysosomes via capturing of heat shock protein 70, an important mediator of cross-presentation (75, 76). This limits cross-presentation and thereby priming of cytotoxic T cell responses (Table 1). Given the importance of FAO in supporting a tolerogenic phenotype by DCs it is tempting to speculate that perhaps oxidation of these lipids in the mitochondria contributes to DC tolerogenicity within the TME (Table 1) (18, 58). What signals trigger the initial increase in lipid uptake by DCs in the TME remains to be determined.

### Hypoxia

Rapid tumor growth results in poorly vascularized regions where oxygen supply is limited. The main metabolic response to hypoxia is stabilization of HIF1α and subsequent activation of glycolysis, which can also occur independently from HIF1α activation during DC activation (16, 17, 107). This may explain why some studies did not find changes in expression of maturation markers or T cell priming capacity when moDCs were stimulated with LPS after differentiation in a 1% O_2_ hypoxic chamber (Table 1) (84, 89). Also differentiation itself of moDCs is mostly unaffected by hypoxic conditions (84–86, 89). Nonetheless there is also some evidence that 1% oxygen can impair LPS-induced moDC maturation and T cell priming potential (Table 1) (85, 87). In addition, hypoxia impaired migration of *in vitro* cultured LPS-treated moDCs and human primary myeloid DCs. Mouse CD34^+^-derived myeloid DCs injected in the footpad of mice after they were treated with LPS and deferoxamine (DFX), a drug that mimics hypoxia, showed reduced migration to the draining lymph node compared to untreated cells, indicating that hypoxia reduces migration both *in vitro* and *in vivo* (87, 89). Interestingly, enhanced expression of migratory genes was found in immature moDCs cultured under low oxygen conditions (86). This could indicate that the hypoxic TME has immunosuppressive effects via capturing mature DCs and elimination of immature DCs. Interestingly, immature moDCs cultured under hypoxic conditions had increased expression of genes involved in sensing chemotactic signals from pro-inflammatory sites and induced secretion of chemotactic factors that attract neutrophils (84, 85, 87). Additionally, murine myeloid DCs treated with DFX increased local leukocyte infiltration *in vivo* (87). Altogether these data obtained from moDCs, may indicate that an oxygen-poor environment triggers DCs to boost innate rather than adaptive immune responses. However, contrary to human moDCs, murine BMDCs cultured in hypoxic conditions enhanced expression of costimulatory molecules, pro-inflammatory cytokine secretion and T cell proliferation upon LPS stimulation (Table 1) (88). Additional studies are needed to determine whether these discrepancies are due to inherent differences between human and murine DCs in their response to hypoxia or caused by differences in experimental setup. In addition, the metabolic context in which DCs are exposed to hypoxia may also affect how DCs respond. For instance, under nutrient replete conditions hypoxia may not compromise DC function, such as in lymph nodes in which hypoxic region have been described (108). In contrast, under pathological conditions, such as in the TME where hypoxia may be also accompanied by nutrient restriction, hypoxia may have anti-inflammatory effects on DCs. Studies addressing the effects of hypoxia on DC biology particularly *in vivo* during homeostasis as well as in pathological settings are needed to fully understand the role of oxygen availability on DC function *in situ*.

### Oxidative Stress

The main sources of ROS in tumor cells are dysfunctional mitochondria and NADPH oxidases. This is further enhanced by intracellular ROS production of stromal cells, as a consequence of the metabolic alterations within the TME (109, 110). Intracellular ROS production in DCs during an immune response can have both pro-inflammatory and anti-inflammatory effects, via modulation of cross-presentation and of signaling pathways (39, 61, 77, 110–112). In general, extracellular ROS seems to have a pro-inflammatory effect, although data is limited (Table 1). Treatment of immature moDCs with hydrogen peroxide enhanced maturation and their capacity to induce T cell proliferation upon LPS stimulation (90). The inflammatory response of primary DCs to the malaria parasite *Plasmodium falciparum* also increased upon exposure to cells treated with xanthine oxidase, a malaria-induced enzyme that increases extracellular ROS levels (91). However, while transient ROS exposure following DC activation may have pro-inflammatory effects, what the functional consequences are of chronic ROS exposure, a situation DCs presumably have to deal within the TME, remains unclear. Possibly, the highly oxidized lipids that are found in TADCs, are one of the byproducts of chronic ROS exposure (113). Through this mechanism long-term ROS exposure in the TME could lead to impaired DC immunogenicity. Studies addressing the functional consequences of transient vs. chronic ROS exposure as well as of the different types of ROS on DCs, will be required to better define what role tumor-associated ROS and oxidative stress play in DC function in the TME.

### Lactate

Tumor cells are known for the Warburg effect, which goes along with secretion of high levels of lactate. Lactate has a major influence on the immune-priming efficiency of DCs. Lactate derived from tumor spheroids, mesenchymal stromal cells or endogenously produced, affects the differentiation and maturation of moDCs. High concentrations of lactate reduce the differentiation capacity of moDCs, as higher numbers of monocyte like CD14^+^/CD1a^−^ cells were detected at the end of the cultures (92–94). This was accompanied by a lactate-dependent reduction of oxidative phosphorylation, but enhanced respiratory capacity in immature moDCs (94). Also maturation of DCs is affected by high lactate levels, reflected by lower levels of maturation markers, an increase in immunosuppressive cytokine secretion, a decrease in pro-inflammatory cytokine secretion and reduced ability to induce T cell proliferation (92, 93, 95). The latter can be caused by detrimental effects of lactate on cross presentation. Using tumor conditioned Flt3L-DCs stimulated with CpG/PolyI:C and OVA-peptide, it was found that high lactate concentrations accelerate antigen processing via lowering the endosomal pH, resulting in impaired preservation of MHC-I epitopes. Thus, high concentrations of lactate in the local environment of differentiating or maturing DCs induces tolerance in DCs, via altering metabolism and antigen processing (Table 1). Extracellular lactate can mediate its anti-inflammatory function via binding to lactate receptor Gi-protein–coupled receptor 81 (GPR81) as was recently shown in DCs derived from murine mammary gland tumors (114). Alternatively, lactate can enter the cells via monocarboxylate transporter 1 (MCT1) as was shown in moDCs (92). Intracellular lactate may also hamper the immune response via a recently discovered epigenetic modification termed lactylation. In M1 macrophages endogenously produced lactate promoted lactylation of lysine residues, thereby promoting M2-like gene expression (115). Whether histone lactylation is another immunosuppressive feature of lactate in DCs is an interesting question that warrants further study.

### ATP and Adenosine

Whereas during homeostasis extracellular ATP levels are negligible, ATP is highly abundant in the TME where it functions as a signaling molecule that provokes inflammation (55, 116). It has been proposed that diffusion of ATP out of the TME recruits DCs to the TME, given that BDMCs treated with 500 uM ATP increased migratory speed (Table 1) (97, 98). Moreover, extracellular ATP released by tumor cells after chemotherapy can promote anti-tumor immunity via signaling through ATP-receptors P2RX7 on DCs, thereby activating the NLRP3 inflammasome, enhancing IL-1β secretion and boosting CD8^+^ T cell priming (99, 100). In contrast, moDCs co-cultured with acute myeloid leukemia cells treated with chemotherapy drugs displayed increased potency to expand regulatory T cells in an extracellular ATP-dependent manner (Table 1) (101). Hence, there is great value in understanding what factors determine the balance between the pro- and anti-inflammatory effects by extracellular ATP after chemotherapy, as it may be an important predictor for treatment efficacy.

Paradoxically, immunosuppressive nucleoside adenosine, derived from conversion of ATP by membrane-bound ectonucleosides CD39 and CD73, is also abundantly present in the TME (117, 118). Adenosine interacts with four different receptors, of which A2AR and A2BR are most highly expressed on immune cells (119). Irradiation of mouse breast tumors caused upregulation of CD73 expression in tumor cells and increased local adenosine concentrations. Anti-CD73 treatment enhanced cDC1 tumor infiltration, increased the antitumor T cell response and reduced tumor growth (102). Additionally, in mice in which adenosine receptor A2BR was selectively knocked out in CD11c^+^ DCs, the growth of B16-melanoma was delayed, supporting a role for adenosine signaling in rendering DCs immunosuppressive (103). Furthermore, LPS-stimulated BMDCs treated with adenosine analog NECA increased intracellular cAMP levels, which lowered secretion of IL-12 and TNF-α secretion and enhanced IL-10 release via protein kinase A (PKA) and exchange protein directly activated by cAMP (Epac) signaling (104). Overall most studies indicate that extracellular ATP enhances immunogenicity of DCs and anti-tumor immune responses, while adenosine does the opposite (Table 1) (98, 120). Shifting the balance in favor of ATP by blocking CD73, CD39 or adenosine receptors is therefore a promising immunotherapy.

### 2-Hydroxyglutarate

In various tumors the oncometabolite 2-HG accumulates, which in non-malignant tissues is found at low concentrations (54). 2-HG has been shown to contribute to immune suppression in the TME via anti-inflammatory effects on T cells (121, 122). Immature moDCs cultured for 24 h with LPS and 2-HG secreted reduced levels of IL-12, enhanced mitochondrial respiration and lowered lactate secretion, indicating that accumulation of 2-HG affects moDCs via metabolic reprogramming. However, the ability of DCs to induce T cell proliferation remained the same, suggesting that the 2-HG-induced metabolic rearrangement in DCs does not affect their T cell priming capacity (Table 1) (105). However, in this context 2-HG was added simultaneously with the TLR ligand, while in the TME immature DCs may reside in a 2-HG-rich environment without immediate activation. Long-term exposure to 2-HG may have a stronger effect on the immunogenic capacities of DCs, potentially via the changes in gene expression, given that 2-HG affects activity of DNA and histone modifying enzymes, but this remains to be determined (54).

### Concluding Remarks

Thus far the effects of metabolic perturbations characteristic of the TME on DC biology have been primarily studied in *in vitro* systems using moDCs. However, we have still limited knowledge about the real contribution of those metabolic changes on the functional properties of conventional as well as inflammatory DCs residing in the TME *in situ*. Likewise, if and to what extent these different metabolic perturbations interact and synergize to affect the functional properties of DCs remains to be determined. For successful activation of the immune system via DC-based therapy it is important to know how DCs deal with these metabolic rearrangements in the TME. For instance, how do DCs respond to adjuvants in the metabolic context of the TME? Is there a way to make these cells less vulnerable to potential immunosuppressive metabolic cues from the TME? And once out of the immunosuppressive metabolic TME, how quickly can DCs regain immunogenic function, if at all possible? To answer these questions and to gain better understanding of the immunosuppressive effects of the metabolic TME on DCs, in depth characterization of the metabolic TME and DC phenotype in primary tumors will be key.

## Effects of the Metabolic Environment on DCs in Diabetes

### Interplay Between Metabolic Disturbances and Inflammation Leading to Diabetes

Not only nutrient deprivation, but also excessive amounts of nutrients can disturb immune homeostasis and DC function. A well-known example of a disease that is characterized by elevated concentrations of glucose and lipids is diabetes. The two main types of diabetes are type I and type II Diabetes Mellitus (T1D/T2D), both characterized by dysfunctional insulin regulation and subsequent hyperglycemia. While T1D develops as a consequence of an auto-immune reaction against beta-cells, common causes for T2D are aging and obesity. Obesity causes hyperglycemia, elevated levels of free fatty acids, hypoxia, oxidative stress and an imbalance in many other metabolites, hormones and cytokines (123–126). This causes a switch in the composition and phenotype of immune cells in metabolic tissues from an anti-inflammatory to a more pro-inflammatory profile and thereby induces chronic low-grade inflammation, which ultimately drives insulin resistance (127–129). cDCs and pDCs are among the immune cells present in adipose tissue and there is a clear correlation between insulin resistance and number of CD11c^+^ DCs present in adipose tissue (62, 130–132). Moreover, several studies have shown that in response to high-fat diet DCs present in murine adipose tissue transition from Th1- to Th17-priming cells, an inflammatory profile linked to the pathogenesis of diabetes (62, 63, 133).

We will here describe how DCs are affected by the metabolic changes in their environment and focus on hyperglycemia and elevated levels of free fatty acids. Oxidative stress and hypoxia are also major metabolic players in diabetes, but to our knowledge there is no data available looking at the effects of these conditions on DC function in diabetic context (125, 126). Hence, we refer to the previous section for the effects of oxygen deprivation and excessive ROS levels on DC function.

### Hyperglycemia

Decreased insulin secretion by beta-cells and lowered sensitivity to insulin signaling reduces the uptake of glucose by cells, which subsequently results in elevated blood glucose levels. As glucose availability plays a major role in DC activation it is conceivable that this glucose imbalance affects DC function. Several studies addressed the effects of hyperglycemia on primary dendritic cells from blood. Both a reduction and an increase in myeloid and pDC counts in blood of patients with T1D and T2D have been reported (Table 1) (64–67, 71). Reduced counts seems to be stronger in patients with poor glycemic control (66). Pro- and anti-inflammatory cytokine secretion by DCs from diabetic patients was not altered following *ex vivo* TLR stimulation, indicating that high blood glucose levels do not directly affect the function of circulating DCs, but primarily their numbers (65, 68, 71). It should be noted that hyperglycemia is not the only (metabolic) difference in blood from diabetic patients and other factors may also influence DC frequencies and function. Since a study in mice showed that hyperglycemia does not influence CD11c^+^ DC differentiation in the bone marrow, it is unlikely that the decrease in circulating DCs is a consequence of impaired DC generation (69). Instead, lower numbers of circulating DCs are possibly a reflection of enhanced migration of DCs to metabolic tissues, where DCs are known to accumulate and contribute to the low-grade inflammation observed in metabolic tissues of T2D patients. As previously mentioned DCs in obese adipose tissue drive a Th17 inflammatory response (62, 63). Interestingly, moDCs exposed to 5.5, 15, and 30 mM glucose for 24 h increase CD83 and CD86 expression and secretion of IL-6 and IL-12 in a dose-dependent manner (61). IL-6 is involved in Th17 differentiation of naïve T cells and was also found to be highly secreted by CD11c^+^ DCs from obese adipose tissue, suggesting that high glucose levels in adipose tissue may contribute to conditioning DCs for Th17 priming (Table 1) (63, 134). However, *in vivo* data connecting glucose levels to DC function in metabolic tissues is currently lacking. *In vitro* generation of tolerogenic moDCs was less efficient with monocytes derived from T1D patients with poor glycemic control in comparison to patients who maintained glycemic control, supporting the hypothesis that a hyperglycemic environment promotes a more pro-inflammatory profile (135). On the other hand, moDCs derived from T2D donors compared to healthy control donors expressed lower levels of maturation markers (64). Moreover, moDC differentiation in high-glucose medium (25 mM) or media supplemented with sera from hyperglycemic T2D patients reduced the number of moDCs, expression of maturation markers and the capacity to induce T cell proliferation after LPS stimulation. In addition, glucose-rich micro-environments increase ROS production and promote activation of the p38 MAPK and Wnt/b-catenin pathways, which are associated with tolerogenic properties of DCs (70, 136–138). Together these *in vitro* studies may indicate that over-abundance of glucose drives monocyte differentiation toward less-proinflammatory DCs, while differentiated moDCs and potentially CD11c^+^ DCs residing in adipose tissue may become more immunogenic in a hyperglycemic environment (Table 1).

### Free Fatty Acids

A cause and consequence of obesity and insulin resistance in T2D is the release of free fatty acids by expanding fat mass (124, 139). FAs are well-known regulators of the immune response. Polyunsaturated FAs (PUFAs) often have anti-inflammatory effects while many saturated fatty acids (SFAs) serve as pro-inflammatory molecules (140, 141). Examples of the latter are palmitic acid (PA) and stearic acid (SA), which together with unsaturated oleic acid (OA) are among the most abundant dysregulated FFAs in obese and T2D patients (123, 142). Especially PA is known for its pro-inflammatory effects and detrimental role in T2D pathogenesis (143). This is partly caused via its effects on DCs. PA in combination with LPS can enhance Th1-associated inflammation, which is driven by TLR4-dependent activation of the NFκB pathway and ROS in moDCs (77, 78, 144). PA also boosts inflammatory properties of activated BMDCs in a TLR4-independent manner, via inhibition of hexokinase (HK) during the late stages of metabolic reprogramming. This inhibition of HK and thereby glycolysis resulted in enhanced mitochondrial respiration, increased mitochondrial ROS levels, elevated activation of the unfolded protein response (UPR) and subsequent induction of IL-23 expression. UPR-dependent IL-23 expression was also confirmed in mice fed a high fat diet (39). BMDCs derived from obese mice additionally increased IL-1β secretion in a NLRP3 inflammasome-dependent manner following stimulation with PA (79). IL-1β and IL-23 are key cytokines involved in promoting Th17 responses and hence PA is a potential driver of insulin resistance (133). However, a direct causal link between enhanced pro-inflammatory cytokine secretion by DCs and Th17 induction in settings of FA exposure still needs to be established as for instance DCs isolated from human blood displayed a reduced capacity to prime T cell responses upon stimulation with PA, despite having increased IL-1β and TNF secretion (80). In contrast to PA, SA does not seem to affect DC function. SA treatment of LPS-stimulated moDCs did not affect expression of maturation markers nor the capacity to induce T cell proliferation (81). Although data on the effects of SA treatment of DCs is still limited, this appears to be different from what is known for macrophages, where SA and PA have been reported to have a similar pro-inflammatory effect (145, 146). OA is a mono-unsaturated FA that has beneficial effects on insulin resistance. In macrophages, this is partly mediated by counteracting the pro-inflammatory effects of SFA (143). Thus far, there is no data available indicating an anti-inflammatory role for OA in DCs. OA treatment had either no effect, or boosted a pro-inflammatory immune response, but has never been tested in combination with SFA stimulation (78, 80, 82, 83).

The balance in dietary intake of SFAs and PUFAs can have great influence on the clinical outcome of diabetes. A comparison of 6, 12, and 24% of SFA in the diet of mice without changing the total dietary fat contribution had a profound effect on macrophage function and insulin resistance, with 12% SFA as the greatest contributor to inflammation and insulin resistance (147). Human data is however thus far inconclusive about the beneficial effects of PUFA-rich and SFA-poor diets on glycemic control of T2D patients (148). Therefore, studies in humans and mice with a focus on the composition in dietary fat and profiling of DCs in metabolic tissues may tell us whether DCs contribute to PUFA-mediated protection against diabetes and/or SFA-mediated development of diabetes, potentially via a PA-induced Th17 response.

In conclusion, compared to other immune cells, there is still little known about the effects on DCs of the FFAs that are most abundant in obesity and T2D patients. While PA stimulation of DCs seems to have the expected pro-inflammatory effects, it is remains unclear if SA and OA influence insulin resistance via DCs (Table 1).

### Concluding Remarks

T1D is characterized by an active immune response against beta-cells, while T2D is associated with chronic low-grade inflammation. Hence, it is perhaps somewhat surprising that *ex vivo* data indicate that hyperglycemia has minimal effect on the function of DCs, that most *in vitro* studies describe a tolerogenic effect of excessive glucose levels on DC differentiation, and that only *in vitro* mature DCs are likely to become more pro-inflammatory. Although currently it cannot be excluded that the latter observations may be due the use of *in vitro* model systems, such as *in vitro* generated moDCs that possibly cannot sufficiently mimic the metabolic alterations and glucose-rich environment that cDCs and pDCs are exposed to *in vivo*, it may in fact indicate that hyperglycemia is not a major driver of the pro-inflammatory profiles of DCs observed in diabetes and that other metabolic and/or immunological cues are more important (62, 130–132). This idea would be consistent with the fact that hyperglycemia is generally associated with impaired immune response against for example infections and tumors (149, 150). A better understanding of how overabundance of various nutrients may act in concert to modulate DC function and to thereby contribute to local inflammation in the context of diabetes will be important for identification of the pathways that lead to inflammation-driven insulin resistance that could be targeted to control diabetes.

## Perspectives and Outlook

It is becoming evident that the metabolic micro-environment has a major influence on DC function and that disturbance of metabolic homeostasis can impact immune responses. We aimed to provide an overview of key metabolites that influence DC phenotype and function. Cancer and diabetes are examples of highly prevalent disorders in which metabolic homeostasis is disturbed, but many more pathologies are associated with dysregulated metabolism. Eating disorders alter nutrient availability, organ-specific pathologies, such as hepatic steatosis impair systemic metabolism and oxidative stress is associated with many diseases including atherosclerosis, cardiovascular diseases and neurodegenerative disorders. Hence, understanding the impact of nutrient availability on the function of immune cells, including DCs, is relevant for a broad range of diseases. Reprogramming the metabolic state of DCs by intervening with nutrient availability can be an effective way to control inflammation. This could be achieved by systemic approaches, including nutritional interventions which are commonly used to control inflammation (151). For instance, lowering caloric intake, by reducing fat and glucose content, can improve glycemic control and subsequently reduce diabetes-associated low-grade inflammation (152–154). Given the pro-inflammatory effects of high glucose and SFA concentration on tissue-associated DCs, it is reasonable to assume that dietary interventions that lead to normalization of glucose and SFA concentrations in the tissue that these cells reside in, will render them less pro-inflammatory, thereby contributing to reduction of local tissue inflammation and eventually improvement of metabolic homeostasis. Alternatively, molecular approaches that directly target cellular nutrient uptake or bioenergetic pathways can make DCs potentially less vulnerable to extracellular nutritional changes and interventions that target energy-sensing enzymes like AMPK can also control inflammation (155). In addition, targeting metabolism of non-immune cells can also have a beneficial effect on the metabolic micro-environment of DCs. For example, therapies that interfere with cancer metabolism to directly impair tumor growth could also have indirect anti-tumor effects by potentially creating a TME with higher nutrient availability that would be more permissive to effective anti-tumor immune responses (156).

Current studies addressing the effects of the metabolic micro-environment on DCs are mostly performed *in vitro* using human moDCs or murine BMDCs. While these studies have provided us important new insights into how nutrient availability can shape DC function, *in vitro* culture conditions often do not fully mimic the complexity and concentrations of various nutrients and metabolites these cells are exposed to *in situ*. For instance, *In vitro*-generated DCs are commonly cultured in media supplemented with 10% fetal calf serum (FCS) and glutamine. Serum is a source for lipids, vitamins, hormones, growth factors, and other compounds, but the exact amounts of these compounds are unknown, differ per batch and may not correspond with concentrations found in tissues that DCs reside in Yao and Asayama (157). Moreover, commonly used culture media, such as RPMI 1640 and DMEM contain supraphysiological levels of glucose (11 and 25 mM, respectively, vs. ~5.5 mM *in situ*) and lower levels of electrolytes including calcium and magnesium (158). The effects of nutrient availability on the function of DC subsets in a more physiological environment, with other metabolic and non-metabolic immunomodulatory signals around, need to be further evaluated, to be able to better assess what the *in vivo* contribution of the metabolic micro-environment is on the functional properties of DCs. To tackle this issue, there have been recent efforts to develop human plasma-like, physiological medium, which contains components, such as amino acids, metabolites, salt ions, and vitamins that are absent from standard media and holds physiologically relevant concentrations of common media components. To minimize the effects of FCS-derived components, medium can be supplemented with either a low percentage (2.5%) of FCS or dialyzed FCS (159–161). Studies using these media found enhanced *in vitro* T cell activation and increased biological similarity between cultured breast cancer cells and primary mammary tumors, providing promising first evidence that these types of media could be used to better mimic physiological setting *in vitro* than classically used culture media (160, 161). These tools will likely also be key to further the field of DC metabolism and to better delineate the interplay between DC function and extra- or intracellular metabolism. In addition, various novel mass-spectrometry, high dimensional flow cytometry and transcriptomics platforms have been developed in recent years that enable one to assess metabolic profiles in tissues at high spatial resolution as well as to characterize metabolic and immunological phenotypes of immune cells present in those tissues at the single cell level. These novel techniques will no doubt greatly improve our understanding of how nutrients shape DC function *in situ*.

Even though many open questions remain, recent work has revealed profound effects of the metabolic micro-environment on DC function in health and disease, which may pave the way for developing DC metabolism-based approaches to treat metabolic and inflammatory disorders.

## Author Contributions

EB wrote the manuscript and prepared the figure. BE supervised and wrote the manuscript. All authors contributed to the article and approved the submitted version.

## Conflict of Interest

The authors declare that the research was conducted in the absence of any commercial or financial relationships that could be construed as a potential conflict of interest.
